# Impact of fluid challenge increase in cardiac output on the relationship between systemic and cerebral hemodynamics in severe sepsis compared to brain injury and controls

**DOI:** 10.1186/s13613-018-0419-1

**Published:** 2018-06-28

**Authors:** Matthieu Le Dorze, Florian Huché, Clément Coelembier, Christophe Rabuel, Didier Payen

**Affiliations:** 1 Department of Anesthesiology and Critical Care, Lariboisière Hospital, APHP, 2 Rue Ambroise Paré, 75010 Paris, France; 2 UMR INSERM 1160, University Paris 7 Denis Diderot, Paris, France

**Keywords:** Brain perfusion, Systemic inflammation, Fluid challenge, Sepsis, Transcranial Doppler

## Abstract

**Background:**

Cognitive dysfunction and delirium after ICU are frequent and may partially result from brain ischemia episodes. We hypothesized that systemic inflammation (severe sepsis or septic shock) modifies the control of brain circulation and the relation between systemic and cerebral hemodynamic after a positive response to fluid challenge (FC).

**Methods:**

Three groups of patients were studied if they increased stroke volume (SV) > 10% after 250 or 500 ml of crystalloids: *control group*: patients free of comorbidity anesthetized for orthopedic surgery; *sepsis group*: patients with severe sepsis or septic shock (classic definition); *brain injury* (*BI*) *group*: trauma brain jury or hemorrhagic stroke with no detectable systemic inflammation. The measurements before and after FC were mean arterial blood pressure (MAP) (radial catheter); SV and cardiac output (CO; transesophageal Doppler); bilateral middle cerebral artery (MCAv) velocity with peak systolic (PSV) and end diastolic (EDV) values (transcranial Doppler); end-tidal CO_2_. The role of MAP increase was investigated by an arbitrarily threshold increase of 5%, called responder in CO and MAP (RR). The remaining patients were call responders in CO and non-responders in MAP (RnR). Nonparametric tests were used for statistical analysis.

**Results:**

Among the 86 screened patients, 66 have completed the protocol: 17 in control group; 38 in sepsis group; and 11 in BI group. All patients increased SV > 10% after FC. Only the sepsis group increased MAP [+ 12 (2–25%), *p *< 0.05] with a significant increase in PSV and EDV [(17 (3–30)% and 17 (12–42)%, respectively (*p *< 0.05)], which did not change in the two other groups. The septic RR or RnR had similar variations in MCAv after FC. The baseline MAP < or > baseline median MAP had similar MCAv.

**Conclusions:**

After a FC-induced increase in SV, MCAv (PSV and EDV) increased only in septic group, mostly independently from MAP increase and from baseline MAP level. Cerebral perfusion becomes passively dependent on systemic blood flow, suggesting a modification of the control of cerebrovascular tone in sepsis-induced systemic inflammation. This information has been considered in the clinical management of septic patients.

**Electronic supplementary material:**

The online version of this article (10.1186/s13613-018-0419-1) contains supplementary material, which is available to authorized users.

## Background

The physiology of cerebral circulation indicates that brain metabolic requirements are primarily covered by cerebral oxygen delivery, i.e., cerebral blood flow (CBF). These stimuli modify local vascular tone at a given cerebral oxygen consumption: (1) autoregulation of CBF when blood pressure (BP) varies; (2) carbon dioxide variations inducing changes in brain vessel caliber, e.g., dilatation in acute hypercapnia and constriction in acute hypocapnia; and (3) oxygen content inducing vascular dilatation in hypoxia and a moderate constriction in hyperoxia. The cerebral specificity for this tuning of vascular tone allows the maintenance of CBF adaptation to metabolic demand with relative independence from systemic hemodynamic changes and their regulatory factors [1–3]. The paucity of data available on the relationship between systemic blood flow (cardiac output CO and stroke volume SV) and CBF in humans [4–8], particularly in presence of systemic inflammation [9, 10] or brain injury [11–14], had motivated this study. The actual debate on fluid resuscitation for patients with acute systemic inflammation associated not with vasopressors is poorly focused on regional blood flow. The present study on human beings aimed to: (1) investigate the modifications of cerebral blood flow velocities as a surrogate of CBF [15] induced by a calibrated fluid challenge increasing CO and SV; (2) evaluate the impact of a sepsis-induced systemic inflammation on the CO/blood flow velocities relationship; (3) compare with patient having brain injury but with non-detectable systemic inflammation.

## Methods

This prospective study was performed in our 20-bed intensive care unit (Lariboisière University Hospital, Paris, France) after receiving ethical approval from the Ethics Committee of the French Society of Intensive Care (CE SRLF 13-43, 2013).

### Patients and fluid loading tests

Eighty-three sedated and mechanically ventilated patients were screened for this study. The “control group” had 17 patients without comorbidity anesthetized for scheduled non-major orthopedic surgery. The measurements were performed 15 min after general anesthesia and intubation when circulatory conditions were stable. The “sepsis group” had 52 patients who met the criteria of severe sepsis and septic shock according to the classic consensus definition [16]. The “brain injury (BI) group” consisted in 14 patients having traumatic brain injury (TBI) or hemorrhagic stroke, with no clinical or biological markers of SIRS and no secondary complications that may induce systemic inflammation. When abnormal markers were present, the patients were excluded (*n* = 3) from the study. Even a modest systemic inflammation cannot be ruled out; no patients had a major systemic inflammation as observed in sepsis patients. For all groups, great care was taken as much as possible to limit the confounding factors, such as abnormal arterial CO_2_ levels, vasoactive drugs or sedation heterogeneity, central temperature, and natremia levels. Ventilation was set to maintain normocapnia, which was continuously monitored using end-tidal carbon dioxide partial pressure (ETCO_2_). The following parameters were collected: ECG, oxygen saturation, CO and SV measurements using transesophageal Doppler (CardioQ^®^, Deltex, UK) [17]; invasive systolic, diastolic, and mean arterial pressures (SAP, DAP, and MAP in mmHg) (radial or femoral catheter) were measured in sepsis and brain injury groups and noninvasively in the control group.

When a fluid challenge (FC) was decided by the senior in charge, it was calibrated to investigate the relationship between systemic and cerebral hemodynamics. The FC consisted in a rapid infusion of 250–500 ml of crystalloids over 5–10 min. Data were collected exclusively in patients who exhibited a positive FC response defined as an increase in SV of at least 10%. Among them, those who exhibited an increase in MAP above an arbitrary limit of 5% were called responders–responders (RR = SV increase > 10% + MAP increase > 5%). When MAP increased less than 5%, these patients were called responders–non-responders (RnR = SV increase > 10% + MAP increase < 5%). Following exclusion criteria were used: increase in SV < 10%; age < 18 years; pregnancy; unstable hemodynamics or absence of sinus rhythm; absence of a window for TCD measurements; previous diagnosis of neurological or psychiatric disorders.

### Data collection

The collected clinical characteristics were: age, gender, administration of sedative and vasopressor agents, Charlson comorbidity score, cardiovascular comorbidities especially hypertension, reason for admission, origin of sepsis, severity of the critical illness assessed by SAPSII score at admission, SOFA score at inclusion. In addition, ETCO_2_ (mmHg), PaCO_2_ (mmHg), natremia (mmol l^−1^), glycemia (mmol l^−1^), and temperature (°C) were monitored and collected during the protocol time. The investigator obtained the following hemodynamic parameters just before and after the FC: SAP, DAP, MAP (mmHg), heart rate (HR, min^−1^), CO (l min^−1^), and SV (ml/b). The trained investigators measured the transcranial Doppler blood flow velocities in both side middle cerebral arteries (MCAv) using pulsed Doppler velocimeter (Transcranial Doppler, Athys^®^, France Lyon). Measurements were manually done avoiding the use of a helmet to obtain the best signal possible. The 2-MHz probe was positioned at the temporal window of the skull checking for the best signal. The side of the skull with the best signal for MCAv measurements was selected for analysis in patients without brain injury (control and septic). The parameters were obtained before FC and repeated immediately at the end of FC at the same site. For the BI group, measurements were performed on the non-lesioned side if lesions were asymmetric. At each time of the protocol, at least three measurements were performed and the highest value was used for analysis. Only two physicians performed the TCD measurements to limit inter-individual measurement variability. Peak systolic (PSV) and end diastolic (EDV) blood flow velocities in MCA were collected.

### Statistical analysis

Statistical analysis was performed using Prism (GraphPad Software, USA). Quantitative variables are expressed as medians (25–75 percentiles). Nonparametric statistical tests were used for continuous variables as Wilcoxon test for intragroup comparisons, and Kruskal–Wallis test and Mann–Whitney test for intergroup comparisons. Statistical significance was defined as *p *< 0.05.

## Results

### Study population

**Fig. 1:**
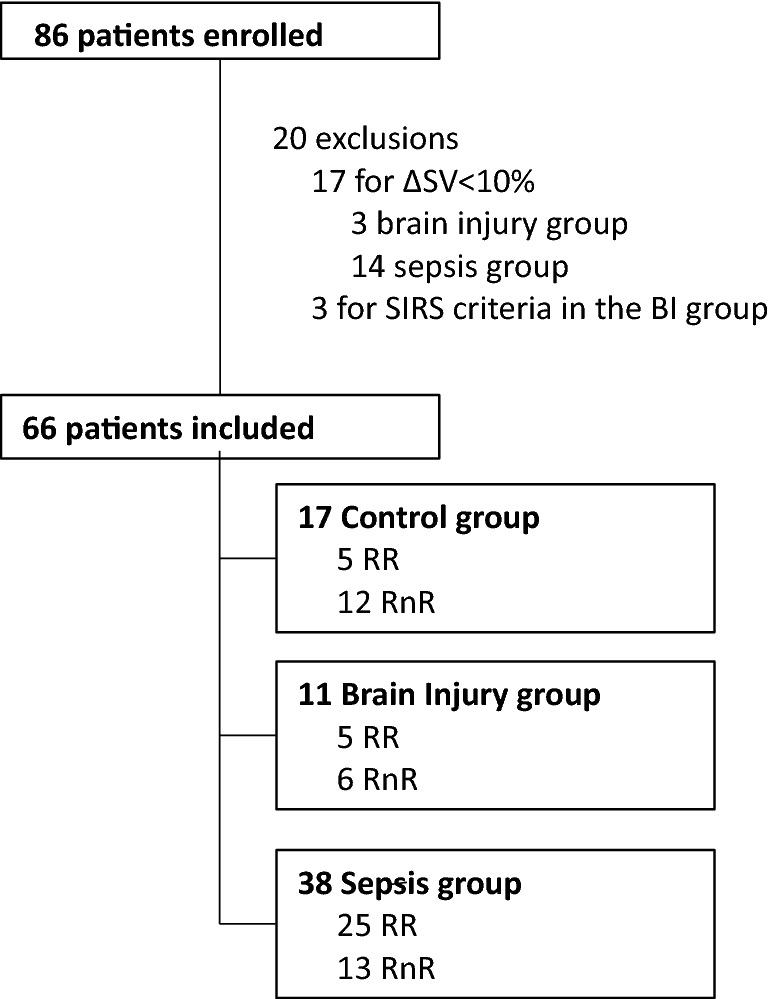
Flowchart of the study. ∆SV: stroke volume variation before and after fluid loading test; RR: responders–responders (∆SV > 10% and ∆MAP > 5%); RnR: responders–non-responders (∆SV > 10% and ∆MAP < 5%)

Figure 1 shows the flowchart of the study population. Among the 86 screened patients, 66 were included. Twenty patients were excluded because of: a SV increase < 10% (*n* = 17) in sepsis and BI groups; three patients had SIRS criteria in BI group. Seventeen patients were in the control group; 38 patients were in the sepsis group, and 11 patients were in the BI group. Table 1 depicts the demographic, clinical characteristics and drugs used for different groups of patients. All sedative or vasopressive drugs (Table 1) remained at the same dose during the protocol. All patients received propofol at a comparable dose ranging (NS): 3.2 mg kg^−1^ h^−1^ in Control, 2.6 mg kg^−1^ h^−1^ in sepsis group, 2.9 mg^−1^ kg^−1^ h^−1^ in BI. All patients were under mechanical ventilation (controlled-assisted ventilation) set to obtain adequate PaCO_2_ and ETCO_2_. 68% (45/66) of patients were measured after 250 ml of crystalloids FC, and 32% (21/66) were measured after 500 ml of crystalloids.

**Table 1:** Demographic characteristics

	Control *n *= 17	Brain injury *n *= 11	Sepsis^a^ *n *= 38 (63% septic shock)
Age (years)	48 (34–59)	58 (41–66)	64 (54–79)
Sexe (female)	12 (70%)	2 (18%)	16 (46%)
Charlson	1 (0–2)	2 (1–4)	3 (2–6)
Hypertension	0	6 (55%)	11 (29%)
Reason for admission	NA	Traumatic 8 (73%)	Abdominal 14 (37%)
		Hemorrhagic stroke 3 (27%)	Pulmonary 10 (27%)
			Skin 7 (18%)
			Other 7 (18%)
SAPS II at admission	NA	49 (42–66)	53 (38–64)
SOFA at inclusion	NA	7 (3–9)	6 (4–11)
Day of FL^b^	NA	0.5 (0–1)	1 (0–3)
MAP (mmHg)	74 (65–89)	89 (77–96)	67 (56–75)
PaCO_2_ (mmHg)	NA	38 (35–42)	39 (36–42)
ETCO_2_ (mmHg)	NA	34 (30–36)	33 (27–35)
Natremia (mmol l^−1^)	NA	138 (133–141)	141 (138–144)
Glycemia (mmol l^−1^)	NA	6.8 (6.8–9.6)	7.3 (5.8–8.6)
Temperature (°C)	NA	37 (37.2–38.0)	37 (36.5–38)
Propofol	17 (100%)	11 (100%)	38 (100%)
Midazolam	0	7 (64%)	11 (29%)
Ketamine	0	0	8 (21%)
Fentanyl	0	9 (82%)	20 (53%)
Norepinephrine	0	7 (64%)^c^	24 (63%)^d^
Epinephrine	0	0	2 (5%)^e^

Continuous values were expressed as median (25th–75th); discontinuous values were expressed with percentage

*SAPSII* Simplified Acute Physiology Score, *SOFA* sequential organ failure assessment, *NA* non available, *FL* fluid loading, *MAP* mean arterial pressure, *ETCO*_*2*_ end-tidal CO_2_

^a^Sepsis group: 14/38 (37%) severe sepsis, 24/38 (63%) septic shock

^b^D0 was the day of admission in ICU

^c^Mean dose 2.3 (0.7–2.8) mg h^−1^

^d^Mean dose 2.8 (0.7–3.5) mg h^−1^

^e^Mean dose 0.25 (0.2–0.29) µg kg^−1^ min^−1^

### Effect of fluid loading test on systemic and cerebral hemodynamic parameters

**Fig. 2:**
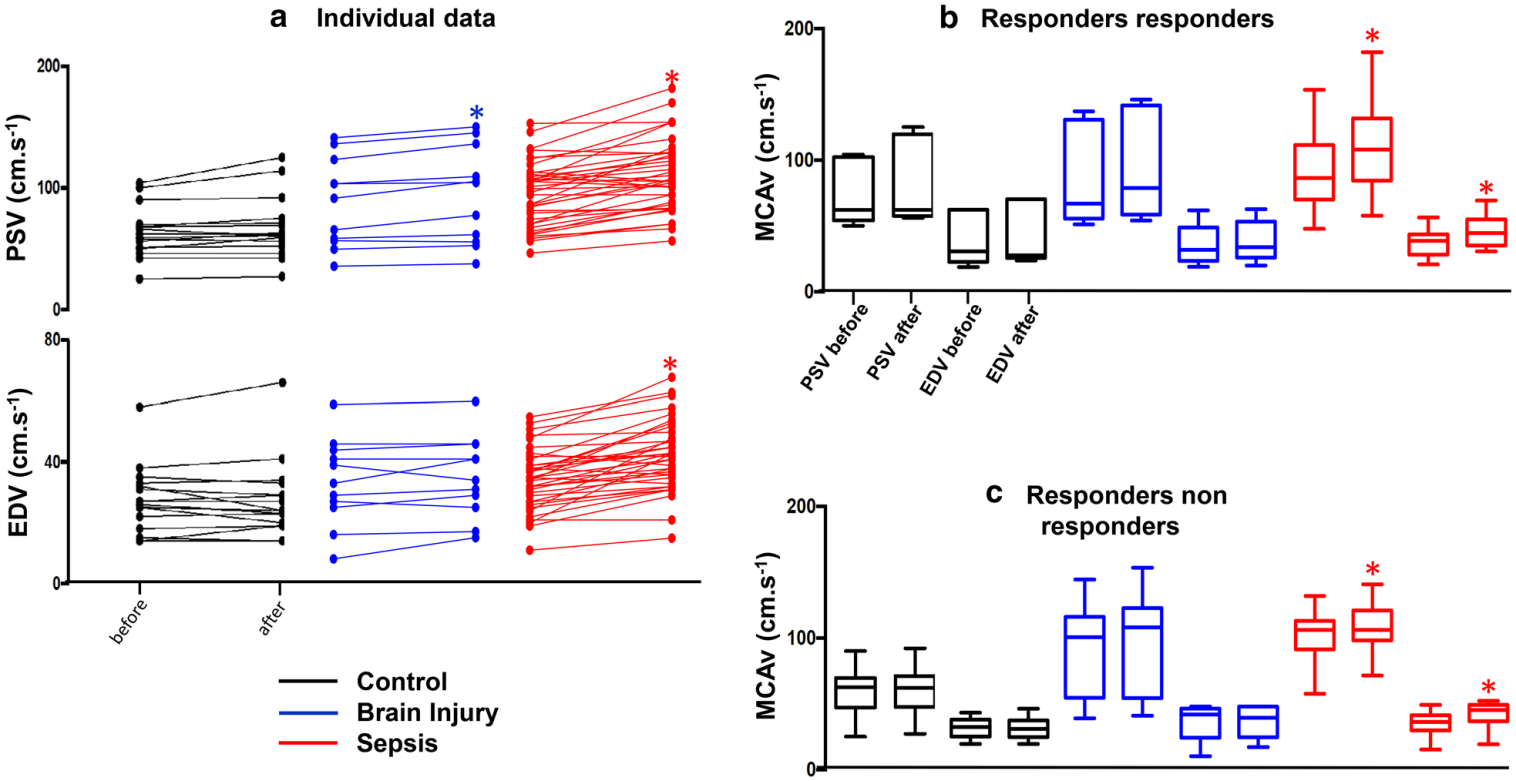
**a** (Left side) The individual data for middle cerebral velocities before and after fluid challenge in the three studied groups: control (black color), brain injury (blue color) and septic patients (red color). The right side of the figure shows the box plot for the middle cerebral artery velocities (MCAv) at peak systole (PSV) and end diastole (EDV); **b** patients responding both in mean blood pressure and CO; **c** patients responding only in CO and not in mean blood pressure. *p value < 0.05

Table 2 shows the baseline values, and the changes in systemic and cerebral hemodynamic parameters for the three studied groups. Figure 2 (left side) shows the individual changes in MCAv. Table 3 shows the baseline values and the changes in systemic and cerebral hemodynamic parameters in sepsis group. Baseline systemic parameters were different between the three groups. Baseline CO and HR were higher in the sepsis group with a lower MAP than the other groups. Since FC was performed with boluses of 250 or 500 ml, the proportion of patients infused with 250 ml was 68%. No Control patients, 40.7% in Sepsis group and 91% of BI group received 250 ml. BI group had a lower CO than in control group with a higher MAP than septic group. Baseline cerebral parameters were also different between the three groups for PSV, which was higher in the sepsis group than in the control group but not with BI group. EDV was comparable between the three groups. Changes in CO, SV, and HR over FC were proportionally similar between the three groups. Arterial blood pressures in the sepsis group (SAP, DAP, MAP) and in the brain injury group (SAP, MAP) changed significantly after the FC. FC induced different changes in cerebral hemodynamic parameters between the three groups (see Additional file 1). PSV and EDV did not change in the control group. Both of them increased in the sepsis group. In BI group, only PSV increased (Table 2 and Fig. 2). When changes were compared to the control (Table 2), changes in EDV (*p* = 0.0001) and PSV (*p* < 0.0042) increased significantly only in sepsis group (Table 3). This increase in sepsis group was also significant for EDV when compared to BI group (*p* = 0.0170). No significant correlations between systemic and cerebral hemodynamic changes were observed in any group (data not shown).

**Table 2:** Systemic and cerebral hemodynamics

	Control *n *= 17	Brain injury *n *= 11	Sepsis *n *= 38	*p*	*p* S versus C	*p* BI versus C	*p* S versus BI
Baseline							
CO (l min^−1^)	4.6 (3.4 to 6.5)	3.2 (2.8 to 3.5)	5.2 (4 to 6.7)	0.0036*	0.03*	0.015*	0.0006*
SV (ml)	66 (54 to 86)	49 (33 to 53)	53 (41 to 72)	0.0087*	0.0302*	0.0023*	0.1188
HR (min^−1^)	72 (62 to 80)	75 (65 to 89)	103 (87 to 123)	< 0.0001*	< 0.0001	0.2485	0.0028*
SAP (mmHg)	105 (96 to 122)	128 (102 to 147)	101 (88 to 113)	0.0146*	0.1995	0.0515	0.0056*
DAP (mmHg)	60 (53 to 75)	66 (60 to 79)	52 (46 to 57)	0.0001*	0.0036*	0.2890	< 0.0001*
MAP (mmHg)	74 (65 to 89)	89 (77 to 96)	67 (56 to 75)	0.0007*	0.024*	0.0926	0.0002*
PP (mmHg)	45 (37 to 51)	54 (40 to 78)	47 (36 to 62)	0.2404			
PSV (cm s^−1^)	62 (51 to 70)	90 (55 to 122)	94 (66 to 107)	0.0068*	0.0006*	0.1950	0.6405
EDV (cm s^−1^)	27 (20 to 34)	32 (24 to 43)	34 (26 to 39)	0.3031			
Fluid challenge							
ΔCO (%)	19 (13 to 31)*	22 (15 to 27)*	27 (17 to 33)*	0.2071			
ΔSV (%)	26 (17 to 46)*	30 (24 to 33)*	27 (19 to 42)*	0.9671			
ΔHR (%)	− 6 (− 12 to 1)*	− 6 (− 8 to − 1)*	− 3 (− 8 to 0)*	0.2682			
ΔSAP (%)	2 (− 2 to 5)	8 (3 to 27)*	12 (4 to 37)*	0.0022*	0.0006*	0.0278*	0.2935
ΔDAP (%)	1 (− 6 to 8)	4 (− 2 to 27)	7 (0 to 14)*	0.1814			
ΔMAP (%)	1 (− 4 to 5)	4 (1 to 13)*	12 (2 to 25)*	0.0026*	0.0007*	0.0511	0.1802
ΔPP (%)	2 (− 6 to 7)	15 (2 to 29)*	22 (5 to 59)*	0.0026*	0.0005*	0.0904	0.2008
ΔPSV (%)	2 (0 to 11)	6 (5 to 11)*	17 (3 to 30)*	0.0075*	0.0042*	0.2789	0.0552
ΔEDV (%)	0 (− 7 to 8)	5 (0 to 16)	17 (12 to 42)*	< 0.0001*	< 0.0001*	0.3461	0.0170

Quantitative values were expressed as median (25th–75th). Statistical tests: nonparametric tests, intragroup comparisons by Wilcoxon, intergroup by Kruskal–Wallis and Mann–Whitney test

*CO* cardiac output, *SV* stroke volume, *HR* heart rate, *SAP* systolic arterial pressure, *DAP* diastolic arterial pressure, *MAP* mean arterial pressure, *PP* pulse pressure, *PSV* pic systolic velocity, *EDV* end diastolic velocity, *C* Control group, *S* septic group, *BI* brain injury group

**Table 3:** Systemic and cerebral hemodynamics in patients responders–responders (RR) versus responders–non-responders (RnR) in the sepsis group

	RR *n *= 23	RNR *n *= 15	*p*
Baseline			
CO (l min^−1^)	5.7 (4 to 6.8)	4.9 (3.5 to 6.7)	0.4288
SV (ml)	54 (38 to 71)	52 (41 to 75)	0.8076
HR (min^−1^)	108 (89 to 124)	93 (71 to 101)	0.0374
SAP (mmHg)	99 (88 to 110)	107 (86 to 121)	0.2382
DAP (mmHg)	51 (44 to 58)	53 (50 to 57)	0.5107
MAP (mmHg)	64 56 to 74)	70 (62 to 77)	0.1685
PP (mmHg)	45 (34 to 60)	49 (42 to 69)	0.2382
PSV (cm s^−1^)	81 (63 to 108)	101 (82 to 107)	0.5205
EDV (cm s^−1^)	36 (25 to 42)	33 (26 to 36)	0.4287
FC test			
∆CO (%)	27 (18 to 35)*	27 (14 to 29)*	0.3323
∆SV (%)	39 (20 to 44)*	25 (14 to 29)*	0.0993
∆HR (%)	− 5 (− 9 to 0)*	− 2 (− 4 to 3)	0.0928
∆SAP (%)	30 (14 to 39)*	1 (− 1 to 5)	< 0.0001
∆DAP (%)	12 (7 to 18)*	0 (− 4 to 3)	< 0.0001
∆MAP (%)	25 (13 to 29)*	0 (− 4 to 4)	< 0.0001
∆PP (%)	52 (22 to 67)*	4 (− 1 to 14)	< 0.0001
∆PSV (%)	24 (11 to 30)*	10 (− 2 to 33)*	0.1348
∆EDV (%)	17 (14 to 42)*	18 (5 to 42)*	0.7400

Quantitative values were expressed as median (25th–75th). Statistical tests: intragroup: nonparametric Wilcoxon test; intergroup: nonparametric Mann–Whitney test

*CO* cardiac output, *SV* stroke volume, *HR* heart rate, *SAP* systolic arterial pressure, *DAP* diastolic arterial pressure, *MAP* mean arterial pressure, *PP* pulse pressure, *PSV* pic systolic velocity, *EDV* end diastolic velocity, *FC* fluid challenge, *RR* responders responders, *RNR* responders non-responders

*Significant difference after FL compared with baseline

### Effects of FC test in RR versus RnR patients

Table 3 and right side of Fig. 2 as the Additional file 1: Table S1 and S2 show the baseline and changes in systemic and cerebral hemodynamics in RR versus RnR patients, especially in sepsis group. The amplitude of changes in PSV and EDV in the sepsis group did not differ between the RR and RnR subgroups, with a noted nonsignificant trend of a higher ΔPSV in RR [24 (11–30)%] than RnR [10 (− 2 to 33)%]. This trend was not observed for EDV, which increased in a same extend. Neither PSV nor EDV were altered after FC in the RR or RnR in the control and BI groups. The control group exhibited a trend of increased EDV in the RR [14 (− 4 to 25)%] versus RnR subgroups [− 2 (− 7 to 5)%], *p* = 0.0764).

## Discussion

Cerebral circulation is regulated by integrated efficient mechanisms, including cerebral autoregulation, hemodynamic-metabolic coupling, and vascular reactivity, to CO_2_ or O_2_ [1–3]. The occurrence of severe sepsis or septic shock requires intense supportive therapy that primarily targets the cardiovascular system using fluid loading and vasopressors [18]. The acute changes in vascular reactivity during acute inflammation [19] induce modifications in regional vascular reactivity, especially at the cerebral level [9]. An improved understanding of the potential modifications of the relationship between systemic and cerebral hemodynamics is of paramount importance because of the post-ICU cognitive dysfunctions and risk of brain ischemia [16]. Relatively few human studies reported the impact of FC on cerebral hemodynamics, especially during systemic acute inflammation. Little is known about human regional circulation because of technical limitations in regional measurements [20]. Regional circulations exhibit different physiological characteristics, with pressure dependence for some circuits and pressure independence for other circuits. Therefore, knowledge of modifications in the autoregulation of flow during pressure variations is necessary, particularly for the brain.

This study hypothesized that the relationship between the systemic circulation and brain perfusion could change in the presence of severe systemic inflammation as in severe sepsis or septic shock, or when local inflammation is present as in brain injury. Such potential modifications can be demonstrated only when it is compared to healthy people under similar sedation. If this hypothesis is correct, such aspect has to be taken into account for therapeutic supportive strategy. The comparison with brain injury patients in the absence of systemic markers of SIRS but with local inflammation was important. This local inflammation may modify the systemic and cerebral circulation responses to FC. The sedation was kept constant during the protocol reducing brain metabolism with a controlled partial pressure of carbon dioxide and adequate arterial oxygen saturation. As a consequence, the impact of these confounding factors controlling cerebrovascular tone was limited.

To our knowledge, this study is the first report to investigate the dynamic relationships between CO, MAP, and MCAv before and after an FC test. The following main results were demonstrated: (1) the increase in SV (and CO) after FC increased systolic and diastolic MCAv only in patients exhibiting sepsis-induced severe acute systemic inflammation; (2) the impact of a systemic blood flow increase on cerebral flow velocities predominated the role of MAP rise in severe acute systemic inflammation; and (3) this hemodynamic response was specific to systemic inflammation because brain injury patients did not exhibit modified MCAv despite the FC-induced MAP increase.

The FC-induced increase in CO does not increase blood pressure in control or BI groups, which confirms the tight control of blood pressure by an induced vasodilatation when CO increases. More specifically, MCAv did not change significantly, with a modest but significant increase (6%) in PSV in BI patients. A causal relationship between CBF and CO was demonstrated in healthy volunteers, whose central blood volume decreased via lower negative pressure [4], standing up [5], albumin infusion [6], or normal saline infusion [7]. Blood pressures remained relatively stable in these studies, and CBF changes secondary to CO changes might result from changes in cerebrovascular resistance. However, the mechanisms responsible for the change in CBF related to changes in CO remain speculative [9, 10]. The present study did not find any relationship between CO changes and MCAv changes in control patients under sedation.

Since the systemic inflammation during sepsis [9, 16] may reasonably include cerebral vessels, one can expect a different control in cerebral vasomotion. Previous studies in septic patients have demonstrated a reduction of CBF and CMRO_2_ during sepsis [21–23]. Some studies demonstrated impaired CO_2_ reactivity [24–26], and other studies reported conserved CO_2_ reactivity [27–29]. Impaired autoregulation was reported [30–32], but other studies observed a maintained autoregulation [27]. Few data on the relationship between CO and CBF during sepsis have been reported [33, 34], especially after a FC. Smith et al. [34] found that carotid blood flow correlated with the cardiac index in 15 patients with septic shock, suggesting a loss of CBF independence in septic patients. However, Straver et al. [33] did not find any correlation between MCAv and cardiac index in 20 patients with septic shock and observed a negative correlation between MCAv and systemic vascular resistance. The baseline higher CO and lower MAP in the sepsis group in our study was associated with a higher PSV, but with an EDV not different than the control group. These observations are consistent with previous studies that demonstrated an increase in MCAv and pulsatile index during the early phase of sepsis [10, 26, 29, 35, 36]. The FC-induced CO increase in the sepsis group of our study was associated with an increased systolic and diastolic BP in some patients and an increased PSV and EDV. This cerebral velocities increase may result from both MAP and CO increase in a relatively hypo-responsive vascular tone.

The RR and RnR subgroups were compared to test the respective roles of blood pressure level and CO increase on the observed MCAv results. The amplitude of MCAv increase after FC in septic patients was similar between the subgroups with or without MAP increase. This lack of difference may result from the predominant vasodilation secondary to inflammation rather than to the vasomotion associated with MAP variations. CO increase induces a CBF increase with a modest impact on inflow pressure. The subtle impact of MAP might be important in the presence of vasodilatation despite the modest increase in pressure. This was approached by the separation of the patients on their baseline values of MAP referred to the median value. The low and higher MAP patients had similar response in term of SPV and EDV increase, suggesting a modest impact of MAP. This reduced vascular response may result from the sepsis-induced vasoparalysis, which is a combination of impaired pressor and dilatator responses secondary to altered endothelial function [37]. A definite conclusion about the mechanism(s) altering the systemic and cerebral circulation needs further specific studies. The observed results during acute severe inflammation may at least warn the clinician of the potential risk of inadequate systemic flow more than pressure control itself. Knowing this relationship may help to reduce the risk of brain ischemia and the incidence of cognitive dysfunction.

There are several limitations to this study. First, the study was not blinded, and the number of patients was relatively small. These results should be confirmed in a larger population using a blinded Doppler measurement. Despite this, we note that our study is the largest cohort reported in septic patients, in addition to a FC test response. If a theoretical limitation related to the operator and his subjectivity for transcranial Doppler measurements is possible, it is limited since tracings were printed for each measure and this limitation may also be present for all groups. Second, transcranial Doppler is a noninvasive and feasible method, but it is only a surrogate of CBF to assess cerebral perfusion, ignoring the vessel diameter. As for many studies, the diameter of the measured cerebral vessel was considered constant, an assumption that might be wrong. This led to interpret the relative changes in MCAv as a good surrogate of relative changes in CBF. The practice of using TCD-MCAv as a CBF surrogate estimation has been accepted for intensive care patients undergoing evaluations of cerebral perfusion, but interpretation must be cautious [38]. Third, the autoregulation capacity was not investigated in this study. A potential impact of the low baseline MAP value in septic group should be kept in mind. This baseline level could be then lower than the lower limit of the autoregulation curve, leading to CBF dependence on MAP. The similar changes in MCAv observed in patients with low and higher MAP in sepsis group suggests however a negligible impact.

## Conclusions

In summary, the increase in SV (and CO) after FC increased systolic and diastolic MCAv only in patients exhibiting sepsis-induced severe acute systemic inflammation. The impact of a systemic blood flow increase on cerebral flow velocities predominates the role of MAP rise in severe acute systemic inflammation.

## Additional file

**Additional file 1: Table S1.** Systemic and cerebral hemodynamics in patients responders-responders (RR) vs responders-non responders (RnR) in the control group. **Table S2.** Systemic and cerebral hemodynamics in patients responders-responders (RR) vs responders-non responders (RnR) in the brain injury group.

## Abbreviations

CBF: cerebral blood flow
CO: cardiac output
DAP: diastolic arterial pressure
EDV: end diastolic velocity
FC: fluid challenge
MAP: mean arterial pressure
MCAv: middle cerebral artery velocity
PSV: peak systolic velocity
RR: responders–responders
RnR: responders–non-responders
SAP: systolic arterial pressure
SV: stroke volume
TCD: transcranial Doppler
