# Supplementary material for: Impact of fluid challenge increase in cardiac output on the relationship between systemic and cerebral hemodynamics in severe sepsis compared to brain injury and controls
Source: Ann Intensive Care. 2018 Jun 28;8:74. doi: 10.1186/s13613-018-0419-1 (PMC6023801; doi:10.1186/s13613-018-0419-1)
Supplement: Supplementary file 1 — Additional file 1: Table S1. Systemic and cerebral hemodynamics in patients responders-responders (RR) vs responders-non responders (RnR) in the control group. Table S2. Systemic and cerebral hemodynamics in patients responders-responders (RR) vs responders-non responders (RnR) in the brain injury group. [file 13613_2018_419_MOESM1_ESM.docx]

| **Table S1.** Systemic and cerebral hemodynamics in patients responders-responders (RR) *vs* responders-non responders (RnR) in the **control group** | | | |
| --- | --- | --- | --- |
|  | RR  n=5 | RNR  n=12 | p |
| Baseline | | | |
| CO (L.min^-1^) | 4.4 (3.8-6.5) | 4.7 (3-6.5) | 0.9696 |
| SV (mL) | 80 (58-101) | 64 (51-75) | 0.2232 |
| HR (.min^-1^) | 64 (57-72) | 74 (69-83) | 0.0868 |
| SAP (mmHg) | 98 (83-115) | 111 (97-125) | 0.1668 |
| DAP (mmHg) | 49 (46-61) | 66 (59-78) | 0.0811 |
| MAP (mmHg) | 62 (59-77) | 82 (70-94) | 0.0676 |
| PP (mmHg) | 48 (37-55) | 44 (37-51) | 0.6290 |
| PSV (cm.sec^-1^) | 62 (54-102) | 63 (47-70) | 0.4273 |
| EDV (cm.sec^-1^) | 26 (18-58) | 27 (20-33) | 0.8188 |
| FL test | | | |
| ∆ CO (%) | 16 (14-35)* | 20 (13-32)* | 0.5356 |
| ∆ SV (%) | 17 (13-40)* | 30 (20-50)* | 0.2605 |
| ∆ HR (%) | -5 (-8--1) | -8 (-15-1) | 0.5052 |
| ∆ SAP (%) | 5 (2-11) | 0 (-3-4) | 0.0637 |
| ∆ DAP (%) | 9 (8-24)* | -1 (-9-2) | 0.0003 |
| ∆ MAP (%) | 5 (5-10)* | -3 (-7-3) | 0.0003 |
| ∆ PP (%) | 0 (-9-1) | 4 (-6-18) | 0.1934 |
| ∆ PSV (%) | 14 (-2-19) | 2 (0-7) | 0.3693 |
| ∆ EDV (%) | 14 (-4-25) | -2 (-7-5) | 0.0764 |
| Quantitative Values were expressed as median (25^th^-75^th^) **Statistical tests**: intragroup: non parametric test by Wilcoxon test; intergroup: non parametric Mann Whitney test. * indicates a significant difference after FL compared with baseline.  CO: Cardiac Output, SV: Stroke Volume, HR: Heart Rate, SAP: Systolic Arterial Pressure, DAP: Diastolic Arterial Pressure, MAP: Mean Arterial Pressure, PP: Pulse Pressure, PSV: Pic Systolic Velocity, EDV: End Diastolic Velocity, FL: Fluid Loading., RR: Responders Responders, RNR: Responders Non Responders | | | |

| **Table S2.** Systemic and cerebral hemodynamics in patients responders-responders (RR) *vs* responders-non responders (RnR) in the **brain injury group** | | | | |
| --- | --- | --- | --- | --- |
|  | RR  n=5 | RNR  n=6 | p | |
| Baseline | | | | |
| CO (L.min^-1^) | 2.9 (2.2-3.4) | 3.2 (3.0-4.7) | 0.3550 | |
| SV (mL) | 49 (27-51) | 48 (31-56) | 0.7013 | |
| HR (.min^-1^) | 71 (60-86) | 83 (69-97) | 0.3203 | |
| SAP (mmHg) | 122 (105-144) | 134 (98-149) | 0.8398 | |
| DAP (mmHg) | 68 (59-81) | 62 (60-70) | 0.3290 | |
| MAP (mmHg) | 89 (72-100) | 88 (75-96) | 0.9999 | |
| PP (mmHg) | 54 (39-70) | 63 (36-88) | 0.5693 | |
| PSV (cm.sec^-1^) | 64 (52-128) | 96 (50-111) | 0.9999 | |
| EDV (cm.sec^-1^) | 28 (19-45) | 39 (21-43) | 0.6277 | |
| FL test | | | | |
| ∆ CO (%) | 15 (12-17)* | 26 (24-33)* | 0.0043 | |
| ∆ SV (%) | 24 (15-34)* | 31 (27-34)* | 0.3203 | |
| ∆ HR (%) | -8 (-12 - -7) | -2 (-5 – 0) | 0.0173 | |
| ∆ SAP (%) | 27 (8-31)* | 4 (-1-9) | 0.0303 |  |
| ∆ DAP (%) | 26 (7-28)* | -1 (-2 – 2) | 0.0087 | |
| ∆ MAP (%) | 13 (11-20)* | 1 (-2 – 3) | 0.0043 | |
| ∆ PP (%) | 29 (0-46) | 9 (2-15) | 0.1255 | |
| ∆ PSV (%) | 7 (6-15) | 5.9 (0.3-8.7) | 0.2273 | |
| ∆ EDV (%) | 7 (4-20) | 0 (-9-28) | 0.1126 | |
| Quantitative Values were expressed as median (25^th^-75^th^). **Statistical tests**: intragroup: non parametric Wilcoxon test; intergroup: non parametric Mann Whitney test. * indicates a significant difference after FL compared with baseline.  CO: Cardiac Output, SV: Stroke Volume, HR: Heart Rate, SAP: Systolic Arterial Pressure, DAP: Diastolic Arterial Pressure, MAP: Mean Arterial Pressure, PP: Pulse Pressure, PSV: Pic Systolic Velocity, EDV: End Diastolic Velocity, FL: Fluid Loading. RR: Responders Responders, RNR: Responders Non Responders. | | | | |
